# Crystal Structure, Infrared Reflection Spectrum, and Improved Microwave Dielectric Characteristics of Ba_4_Sm_28/3_Ti_18_O_54_ Ceramics via One-Step Reaction Sintering

**DOI:** 10.3390/ma17143477

**Published:** 2024-07-13

**Authors:** Zeping Li, Huajian Zhou, Gang Xiong, Huifeng Wang, Geng Wang

**Affiliations:** 1 School of Electronic and Information Engineering, Hubei University of Science and Technology, Xianning 437100, China; 2 Key Laboratory of Photoelectric Sensing and Intelligent Control, Hubei University of Science and Technology, Xianning 437100, China

**Keywords:** Ba_4_Sm_28/3_Ti_18_O_54_ ceramics, microwave dielectric characteristics, reaction sintering, infrared reflection spectrum

## Abstract

High-k Ba_4_Sm_28/3_Ti_18_O_54_ ceramics with improved microwave dielectric characteristics were successfully fabricated using the one-step reaction sintering (RS) route. The sintering characteristics, microstructure, crystal structure, infrared reflection spectrum, and microwave dielectric characteristics of Ba_4_Sm_28/3_Ti_18_O_54_ ceramics prepared by the RS route were systematically investigated. Samples prepared by the RS route exhibited single-phase orthorhombic tungsten–bronze structure and dense microstructure at optimum sintering temperature. Compared with the conventional solid-state (CS) process, the Ba_4_Sm_28/3_Ti_18_O_54_ ceramics fabricated by the RS route presented a smaller temperature coefficient (TCF), a higher quality factor (*Q × f*), and a higher permittivity (*ε_r_*). The improved microwave dielectric characteristics were highly dependent on the theoretical permittivity, atomic packing fraction, suppression of Ti^3+^, and Ti-site bond valence. Excellent combined microwave dielectric characteristics (TCF = −7.9 ppm/°C, *Q × f* = 9519 GHz, *ε_r_* = 80.26) were achieved for Ba_4_Sm_28/3_Ti_18_O_54_ ceramics prepared by RS route sintered at 1400 °C, suggesting the RS route was a straightforward, economical and effective route to prepare high-performance Ba_4_Sm_28/3_Ti_18_O_54_ ceramics with promising application potential.

## 1. Introduction

Recently, the widespread application of 5G/6G wireless communication techniques has brought increasing requirements for high-performance microwave components (e.g., antennas, duplexers, filters, dielectric resonators, etc.) [1,2,3,4,5]. Being a critical material for the fabrication microwave components, microwave dielectric ceramics have attracted continuing commercial and scientific attention because of their superior properties [6,7,8,9,10]. Generally, high-performance microwave dielectric ceramics are desired to have a small temperature coefficient (*τ_f_* or TCF, here TCF is a measure of the “drift” with respect to the temperature of the resonant frequency) to meet thermal stability, a high quality factor (the quality factor (Q), which is a function of resonant frequency (*f*), is sometimes expressed as *Q × f*) to enable good frequency selectivity for microwave components, and a high permittivity (*ε_r_*) in order to realize miniaturization [11,12,13,14,15]. However, achieving the three dielectric characteristics mentioned above (high *Q × f*, small TCF, and high *ε_r_*) simultaneously in high-k microwave dielectric ceramics for 5G applications is a significant challenge.

The typical high-k microwave dielectric ceramics include BiVO_4_-based, Bi_2_O_3_-Li_2_O-Ta_2_O_5_, lead-based perovskite, SrTiO_3_-based, LiO_2_-Nb_2_O_5_-TiO_2_, Na_2_O-M_2_O_3_-TiO_2_, CaO-LiO_2_-M_2_O_3_-TiO_2_, (Ca_1−*x*_M_2*x*/3_)TiO_3_, and Ba_6−3*x*_M_8+2*x*_Ti_18_O_54_ systems (M = Pr, Sm, La, Nd) [16,17,18,19,20,21,22,23,24,25]. As a member of the high-k microwave ceramic ceramics, tungsten–bronze Ba_6−3*x*_M_8+2*x*_Ti_18_O_54_-based (M = Nd, Pr, Sm) ceramics have been receiving continued attention by the researchers due to the high-k (*ε_r_* > 80) and high quality factor (*Q × f* > 5000 GHz) [26]. Specifically, the Ba_4_Sm_28/3_Ti_18_O_54_ (BST, *x* = 2/3) ceramics with outstanding microwave dielectric characteristics (TCF~−10 ppm/°C, *Q × f*~9000 GHz, and *ε_r_*~80) have been regarded as a suitable material in 5G applications [27]. Ohsato et al. [28] reported that the phase constitution of BST ceramics is refined as an orthorhombic tungsten–bronze phase with Pbnm (No. 62, superlattice) by Rietveld analysis using high-resolution XRD data. Wu et al. [29] studied the Raman spectroscopy of BST ceramics and suggested that the Raman modes of the Pbnm were 5B_2g_ + 5B_3g_ + 7A_g_ + 7B_g_. The combining of different R^3+^ (R = Nd, Pr, La) cations is an efficient approach to modify the dielectric performance in BST ceramics, small TCF, high *ε_r_* and low *tanδ* are obtained through the variation of Sm/Nd, Sm/Pr and Sm/La ratios [30,31,32]. Santha et al. [33] investigated the effect of BaCu(B_2_O_5_) on the the dielectric characteristics and sintering temperature (S.T.) of BST ceramics, and the S.T. of BST ceramics could be lowered to 950 °C with 10 wt% BaCu(B_2_O_5_). Different processes have been developed to produce BST ceramics, such as the sol-gel route, classical solid-state (CS) method, and spark plasma sintering (SPS) process [33,34,35]. These approaches are relatively complicated, and the repetitive synthesis process results in fluctuations in the microwave dielectric characteristics of BST ceramics.

Recently, the one-step reaction sintering (RS) route without calcination and secondary ball-milling has been attracting considerable interest because of its high efficiency and process simplification. Du et al. [36] have successfully prepared AlON transparent ceramics with a transmittance of 73.2% at 1600 nm using one-step RS route and tape-casting technology. Liang et al. [37] reported the piezoelectric properties of Bi_4_Ti_3_O_12_ ceramics could be significantly improved using the RS route and Ta/Mn co-doping. In addition, numerous microwave dielectric ceramics have been successfully fabricated using the RS route, such as BaTi_5_O_11_, Ba_2_Ti_9_O_20_, Li_2_MgTi_3_O_8_, 0.5Ca(Mg_1/3_Nb_2/3_)O_3_-0.5Ca_0.6_La_0.267_TiO_3_, Li_3_Mg_2_NbO_6_, Ca_1.15_M_0.85_Al_0.85_Ti_0.15_O_4_ (M = Y, La, Nd), CaTiO_3_-LnAlO_3_ (Ln = La, Nd), Mg_4_Nb_2_O_9_, MgAl_2_O_4_-CoAl_2_O_4_, AZrNb_2_O_8_(A = Zn, Mg, Co), and Ln_2_Zr_3_(MoO_4_)_9_ (Ln = La, Ce, Nd) [38,39,40,41,42,43,44,45,46,47,48]. Although there have been many studies on tungsten–bronze-structure BST microwave dielectric ceramics, the preparation of BST ceramics by the one-step RS route has not been reported until now.

One problem with using TiO_2_ as a raw material is the reduction of Ti^4+^ to Ti^3+^ in high sintering temperature. This may result in the presence of oxygen vacancies ($V_{O}^{••}$), which are regarded as detrimental to microwave dielectric loss (*tan δ* = 1/*Q*). During the high-temperature sintering process in air, quasi-free electrons and oxygen vacancies ($V_{O}^{••}$) are simultaneously generated in the crystals owing to insufficient oxygen partial pressure [49,50]. F-type color centers can be formed by oxygen vacancies ($V_{O}^{••}$) in combination with weakly bound electrons, which have three forms: F^2+^ ($V_{O}^{••}$, bare oxygen vacancies), F^+^ ($V_{O}^{••}\;e^{\prime }$, oxygen vacancies with one trapped electron), and F ($V_{O}^{••}\;2e^{\prime }$, oxygen vacancies with two trapped electrons) [51,52,53]. The recent efforts to restrain the reduction of Ti^4+^ and oxygen vacancies ($V_{O}^{••}$) have been a significant area of research. Doping is an effective method of reducing dielectric loss, thereby enabling the material to meet the requirements of commercial applications. The dielectric loss of TiO_2_-based microwave dielectric ceramics (TiO_2_, BaTi_4_O_9_, Na_0.5_Sm_0.5_TiO_3_, Ba_2_Ti_9_O_20,_ 0.73ZrTi_2_O_6_-0.27MgNb_2_O_6_, Ba_6−3*x*_Sm_8+2*x*_Ti_18_O_54_ and Ba_6−3*x*_Nd_8+2*x*_Ti_18_O_54_) will be significantly reduced by the introduction of a range of divalent or trivalent acceptor cations with ionic radii between 0.5 Å and 0.95 Å, such as Cr^3+^, Al^3+^, Ga^3+^, Zn^2+^, Mg^2+^, Mn^2+^, Cu^2+^ and Co^2+^ [21,49,50,53,54,55,56,57,58]. It is worth noting whether the dielectric loss of BST ceramics can be improved by RS route.

In this study, the BST ceramics with improved microwave dielectric characteristics prepared by the one-step RS route were first introduced. The effects of various sintering routes (CS and RS routes) on the crystal structure, microstructure, valence states of Ti ions, and microwave dielectric characteristics of the BST ceramics were studied. Based on the Rietveld analysis, the theoretical permittivity (*ε_the_*), atomic packing fraction (P.F.%), and Ti-site bond valence (V_Ti_) were analyzed to study the structural properties of BST ceramics. Furthermore, infrared reflection spectrum was employed to evaluate intrinsic dielectric characteristics of BST ceramics prepared by RS route.

## 2. Materials and Methods

**Figure 1:**
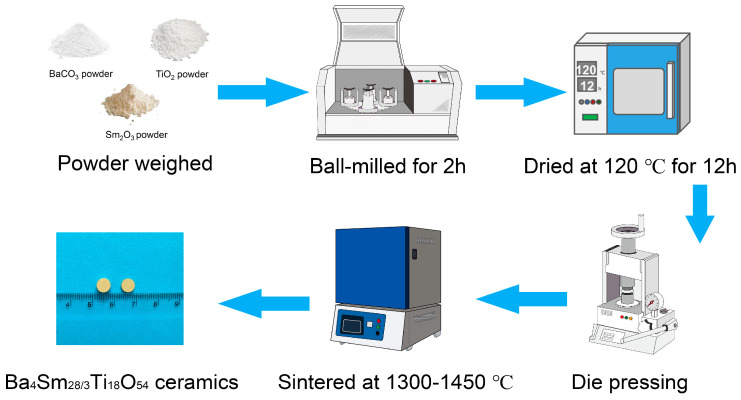
One-step reaction sintering process for BST ceramics.

Ba_4_Sm_28/3_Ti_18_O_54_ ceramics were prepared via one-step RS and CS processes employing high-purity Sm_2_O_3_ (99.99%, Aladdin, Shanghai, China), TiO_2_ (99.84%, Zhongxing, Xiantao, China), and BaCO_3_ (99.8%, Yuanda, Mianyang, China) powders. In particular, Sm_2_O_3_ powder was calcined at 950 °C/4h for moisture removal. The oxide and carbonate powders were then weighted to achieve the desired stoichiometric ratio of Ba_4_Sm_28/3_Ti_18_O_54_ (BST). For the RS process, the blended oxide and carbonate powders were milled in de-ionized water with ZrO_2_ balls (diameter 2~8 mm) for 2 h. After oven drying at 120 °C/12 h, the drying oxide and carbonate powders were combined with the binder (10 wt% PVA). The ground and sieved powders were molded into cylindrical pellets (Φ8 × 5 mm, and Φ8 × 2 mm). For the CS process, the detailed experimental procedure was described in earlier work [5]. All these pellets prepared by RS and CS route were subsequently sintered in an atmospheric environment at 1300 °C/4 h to 1450 °C/4 h. Figure 1 presents the detailed procedure for the preparation of BST ceramics by the RS route due to the absence of calcination and secondary ball-milling, the RS route is a simple and efficient preparation method compared to the CS route.

The phase structure of sintered BST ceramics prepared by RS and CS routes was detected via aX-ray diffractometer (XRD; BRUKER, Billerica, MA, USA, D8 advance). A Scanning Electron Microscope (SEM; ZEISS, Oberkochen, Germany, GEMINI300) was utilized to evaluate the natural sintered surface of the BST samples. An infrared reflection (IR) spectrum (50~4000 cm^−1^) was collected in atmospheric (500~5000 cm^−1^) and vacuum (40~700 cm^−1^) environments using an FTIR spectrometer (BRUKER, USA, IFS 66v). The titanium (Ti) valence state in BST ceramics was analyzed by an X-ray Photoelectron Spectrometer (XPS; KRATOS, Kyoto, Japan, Axis Ultra DLD). Microwave dielectric characteristics (TCF, *ε_r_*, and *Q × f*) of BST ceramics were evaluated at microwave frequency (5–6 GHz) via the Hakki–Coleman technique [59] by a network analyzer (KEYSIGHT, Santa Rosa, CA, USA, E5071C).

## 3. Results and Discussion

**Figure 2:**
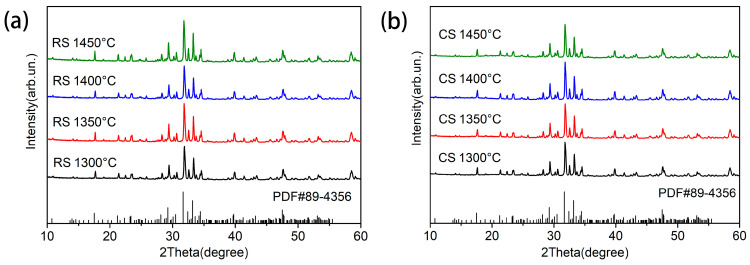
XRD patterns of BST ceramics: (**a**) RS route sintered at 1300–1450 °C; (**b**) CS route sintered at 1300–1450 °C.

**Figure 3:**
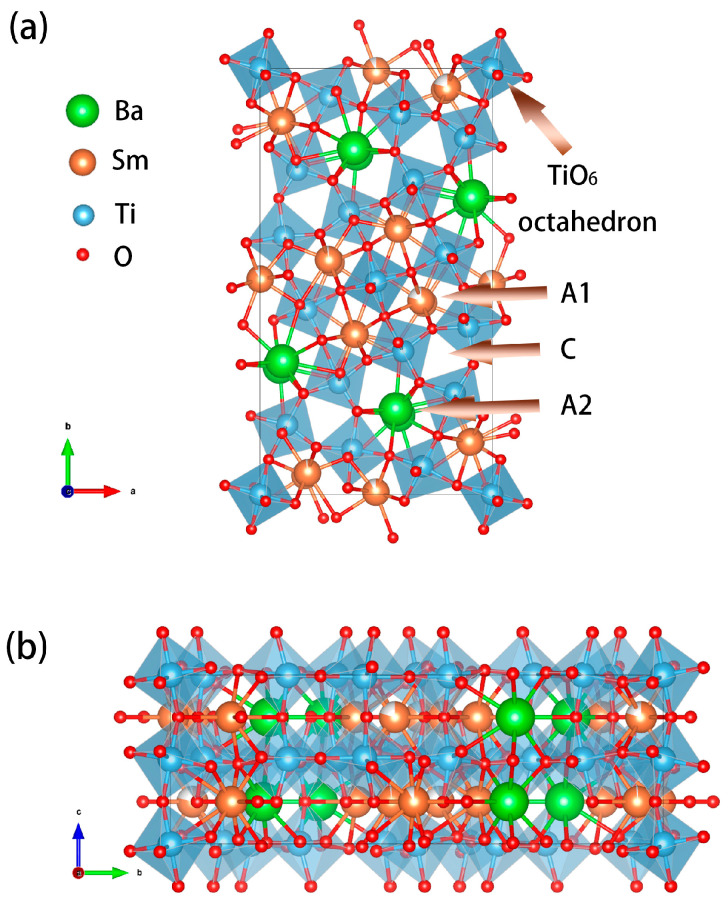
Structural diagrams of BST ceramics projected along (**a**) [001]; (**b**) [100].

The XRD patterns of BST ceramics prepared using one-step RS and CS routes are displayed in Figure 2. As illustrated in Figure 2, all diffraction peaks of BST ceramics prepared by RS and CS routes corresponded to the tungsten–bronze phase (JCPDS No. 89-4356, Ba_3.99_Sm_9.34_Ti_18_O_54_) with orthorhombic structure (Pbnm, No. 62) [28], and no diffraction peaks of impurity phases were detected over the entire sintering temperature range. Structural diagrams of tungsten–bronze-structure BST ceramics with VESTA3 projected along [001] and [100] are presented in Figure 3 [60]. As shown in Figure 3a, the crystal structure of the tungsten–bronze-type BST ceramics consists of a three-dimensional framework of corner-sharing TiO_6_ octahedron linked at the corners to form three types of cavities: diamond cavities (A1), large pentagonal cavities (A2), and small triangular cavities (C). Sm is expected to be located in A1 cavities and larger Ba in the A2 cavities. Small triangular cavities are empty [28].

**Figure 4:**
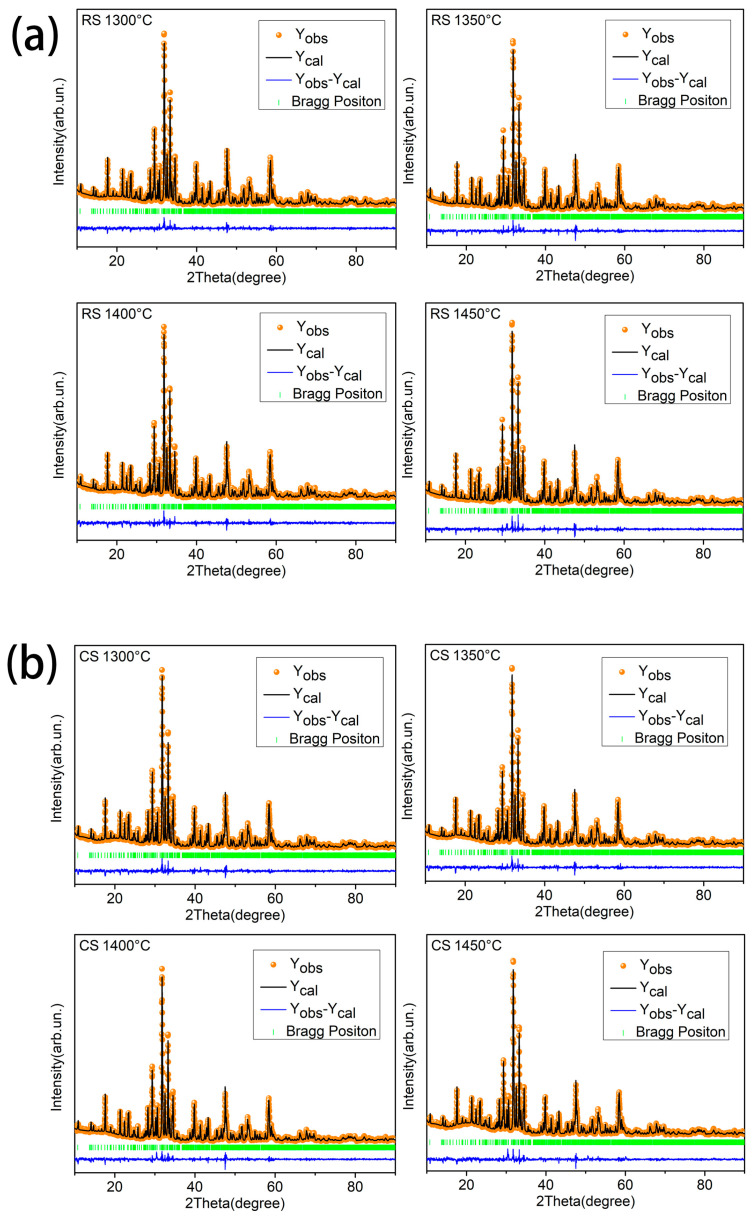
The refinement plots of BST ceramics: (**a**) RS route sintered at 1300–1450 °C; (**b**) CS route sintered at 1300–1450 °C.

To further analyze the influence of various sintering routes and sintering temperatures on the lattice parameters and crystal structure of BST ceramics produced by the CS and RS routes, the Rietveld approach was applied using the GSAS application [61,62]. The fitted (black solid line) and measured (orange dot) XRD profiles of BST samples fabricated using the CS and RS methods are represented in Figure 4, and the fitted XRD were in reasonable accordance with the measurements. Table 1 presents the calculation of structure parameters (a, b, c), cell volume (*V*), theoretical density (*ρ_th_*), and reliability factors (*R_p_*, *χ^2^*, *R_wp_*) of all BST ceramic samples. As illustrated in Table 1 and Figure 4, small reliability factors (*R_p_*, *R_wp_*) of less than 10% were observed, indicating that the results of the refinement were acceptable. It should be noted that the cell volume of BST ceramics produced using the CS route (*V* = 2079.12 Å^3^, *a* = 12.1596 Å, *b* = 22.312 Å, *c* = 7.6633 Å, *R_p_* = 6.00%, *R_wp_* = 8.01%, *χ^2^* = 1.81) is larger than that of the RS route (*V* = 2073.65 Å^3^, *a* = 12.1476 Å, *b* = 22.2878 Å, *c* = 7.659 Å, *R_p_* = 6.03%, *R_wp_* = 7.62%, *χ^2^* = 1.45) at optimal sintering temperature (1400 °C), which is a crucial factor in determining the microwave dielectric characteristics of BST ceramics prepared by different sintering routes.

**Figure 5:**
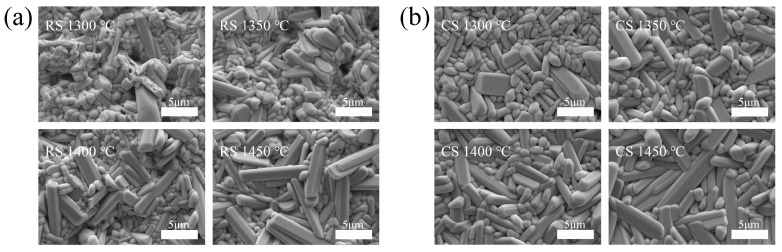
SEM images of BST ceramics: (**a**) RS route sintered at 1300–1450 °C; (**b**) CS route sintered at 1300–1450 °C.

The as-fired SEM photographs of BST ceramics sintered at 1300 to 1450 °C produced by the RS and CS route are presented in Figure 5. As presented in Figure 5, it is clear that all BST samples prepared by the RS and CS routes have rod-shaped grains of tungsten–bronze structural ceramics [25]. The number of rod-shaped grains increases when it is sintered at an increasing temperature from 1300 to 1450 °C. For the CS route, all ceramic samples sintered at different temperatures present a dense microstructure with well-defined grain boundaries, a gradually increasing grain size with increasing temperature is clearly observed, while for the RS route, the samples exhibit a porous and fine-grained microstructure when they are sintered at 1300 °C. For the samples sintered at 1400 °C and 1450 °C, the grain size increases with increasing sintering temperature, while the number of pores decreases significantly, and a relatively dense microstructure with well-defined grain boundaries is observed. Note that the grain size of the BST samples fabricated by the RS route is smaller than that of the CS route at the same temperature, which can be attributed to the absence of a calcination stage in the RS route.

**Figure 6:**
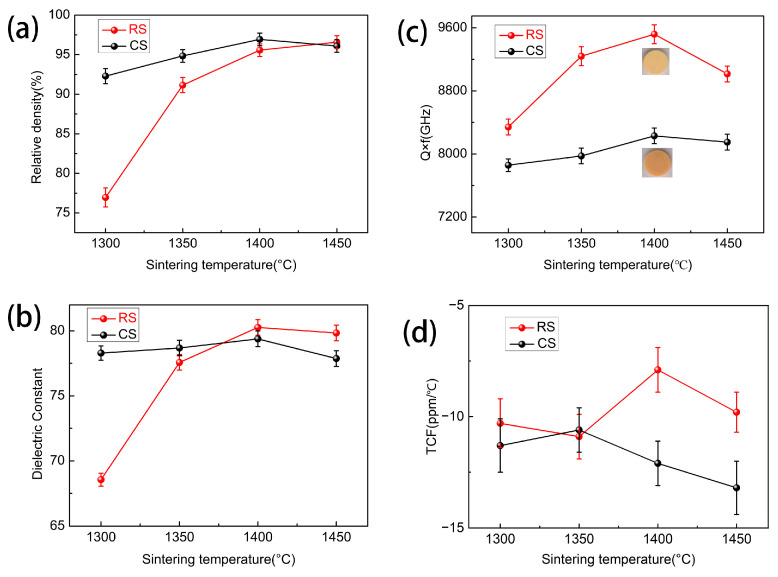
(**a**) Relative densities of BST ceramics; (**b**) *ε_r_* of BST ceramics; (**c**) *Q × f* of BST ceramics (Inserted images show BST ceramics prepared by RS and CS routes sintered at 1400 °C); (**d**) TCF of BST ceramics using RS and CS routes.

Figure 6a presents the relative densities of BST ceramics using RS and CS routes sintered at 1300 to 1450 °C. The relative densities (*ρ_re_*) of BST samples can be evaluated by Equation (1) [47]:

(1) $$\rho_{re}=\frac{\rho_{bu}}{\rho_{th}}\times 100\%$$

where *ρ_re_* and *ρ_bu_* are theoretical density (Table 1) and bulk density of BST ceramics, respectively. As a result of the increase in sintering temperature from 1300 to 1450 °C, the relative density (*ρ_re_*) of the BST samples prepared by CS route exhibits a tendency to increase slowly and then decrease, a relative density of 96.88% was achieved for BST ceramic samples calcined at 1150 °C/4 h and sintered at 1400 °C/4 h. For the RS route, a greater increase in the ρre of BST ceramics was observed for the 1300 °C and 1400 °C temperature ranges, i.e., from a relative density (*ρ_re_*) of 76.96% to 95.57%, indicating that rapid densification should take place at a temperature of over 1300 °C. This result is in accordance with that of SEM (Figure 5a). The improvement in relative density with increased temperature may be associated with porosity reduction.

The permittivity and *Q × f* values of BST samples sintered at 1300 to 1450 °C using RS and CS routes are presented in Figure 6b and Figure 6c, respectively. The trends in permittivity and *Q × f* values of all BST ceramics produced by the CS and RS routes varying with sintering temperature is similar to the relative density (*ρ_re_*). The increased permittivity (*ε_r_*) is associated with a pore (*ε_r_ =* 1) reduction and an increase in relative density, and the improved Q × f values are related to the grain growth and reduction in grain boundaries. The TCF values of BST ceramics fabricated by RS and CS routes are shown in Figure 6d. This study shows that the sintering temperature is not directly related to TCF as a result of no other additional additives and the single-phase BST ceramic samples. Near-zero TCF values (around −10 ppm/°C) were obtained for all ceramic samples prepared by RS and CS routes.

Interestingly, the BST ceramics produced by the RS route have improved microwave dielectric characteristics than those produced using the CS route at the optimum sintering temperature (1400 °C). The microwave dielectric characteristics of BST ceramics prepared by RS and CS routes sintered at 1400 °C are listed in Table 2. Compared to the CS route (*ε_r_* = 79.38, *Q × f* = 8230 GHz, TCF = −12.1 ppm/°C), the BST ceramics fabricated by the RS route present a higher quality factor (*Q × f* = 9519 GHz), a lower dielectric loss (*tanδ* = 6.18 × 10^−4^), a smaller temperature coefficient (TCF = −7.9 ppm/°C), and a higher permittivity (*ε_r_* = 80.26). The RS route is suggested to improve the microwave dielectric characteristics of BST ceramics. To investigate the effect of various preparation processes on the microwave dielectric characteristics of BST ceramics, atomic packing fraction, theoretical permittivity, bond valence, and vacancy defects were analyzed. The results of theoretical permittivity (*ε_the_*), atomic packing fraction (P.F.%), and Ti-site bond valence (V_Ti_) of BST ceramics produced by the CS and RS routes at optimal sintering temperature (1400 °C) are listed in Table 2.

On the basis of previous literature [63,64], the theoretical permittivity (*ε_the_*) can be evaluated using the Clausius–Mossotti (CM) formula (Equation (2)):

(2) $$\varepsilon_{the}=\frac{3V_{m}+8\pi \alpha_{the}}{3V_{m}-4\pi \alpha_{the}}\;$$

where *V_m_ = V/Z* denotes molar volume, and *α_the_* represents theoretical dielectric polarizability. The *α_the_* of Ba_4_Sm_28/3_Ti_18_O_54_ ceramics can be calculated using Equation (3) [63]:

(3) $$\alpha_{\mathrm{the}}({\mathrm{Ba}}_{4}{\mathrm{Sm}}_{28/3}{\mathrm{Ti}}_{18}O_{54})=4\alpha_{\mathrm{the}}\left({\mathrm{Ba}}^{2+}\right)+\frac{28}{3}\alpha_{\mathrm{the}}\left({\mathrm{Sm}}^{3+}\right)+18\alpha_{\mathrm{the}}\left({\mathrm{Ti}}^{4+}\right)+54\alpha_{\mathrm{the}}\left(O^{2-}\right)$$

where α(Ba^2+^) = 6.4 Å^3^, α(O^2−^) = 2.01 Å^3^, α(Ti^4+^) = 2.93 Å^3^, and α(Sm^3+^) = 4.74 Å^3^. According to the CM formula, the theoretical permittivity (*ε_the_*) is inversely proportional to the molar volume (*V_m_*) when the theoretical dielectric polarizability of Ba_4_Sm_28/3_Ti_18_O_54_ ceramics is constant. As listed in Table 1, due to cell volume (*V* = 2073.65 Å^3^) of BST ceramics fabricated by the RS route is smaller than that fabricated by the CS route (*V* = 2079.12 Å^3^), the theoretical permittivity calculated from CM formula of RS route (*ε_the_* = 43.27) is higher than the CS route (*ε_the_* = 41.65), and the tendency of measured permittivity (*ε_r_*) is similar with the theoretical permittivity (*ε_the_*). It is worth noting that the *ε_r_* is much higher than the *ε_the_*, which is attributed to the low symmetry structure of the BST ceramics. The same large deviations are found in other Ti-based microwave dielectric ceramics [30,65,66,67].

A higher *Q × f* value (9519 GHz) for the RS route prepared ceramic sample was achieved compared to a *Q × f* value (8230 GHz) for the CS route is shown in Table 2. Generally speaking, the total *tanδ* (1/*Q*) of functional ceramics is classified into the extrinsic and intrinsic loss. The extrinsic loss is primarily associated with pores, dopants, impure phases, lattice defects, grain boundaries, vacancy defects, etc., while intrinsic loss is primarily attributed to lattice vibration and structural characteristics. In this work, the effects of pores and impure phases on *tanδ* could be ignored as the BST ceramics produced by the CS and RS routes at optimum sintering temperature (1400 °C) with high densities (*ρ_re_* > 95%) and single-phase structure.

As reported by Kim et al. [68], dielectric loss (*tanδ*) is highly associated with structural characteristics of microwave ceramics. The structural characteristics of microwave ceramics could be assessed by the packing fraction, as this parameter is related to the cell volume (*V*) and effective ion size. On the basis of the results of Rietveld analysis, the packing fraction (P.F.%) of the BST ceramics prepared by the RS and CS routes is evaluated by means of Equation (4) [68]:

(4) $$\mathrm{Packing}\;\mathrm{fraction}(\%)=\frac{\mathrm{volume}\;\mathrm{of}\;\mathrm{packed}\;\mathrm{ions}}{\mathrm{volume}\;\mathrm{of}\;\mathrm{unit}\;\mathrm{cell}}\times Z=\frac{\frac{4}{3}\pi \left[4r_{Ba}^{3}+\frac{28}{3}r_{Sm}^{3}+18r_{Ti}^{3}+54r_{O}^{3}\right]}{V}\times 2$$

where V refers to cell volume, $r_{Sm}$, $r_{Ba}$, $r_{Ti}$, and $r_{O}$ are the effective ionic sizes of Sm (1.079 Å), Ba (1.52 Å), Ti (0.605 Å), and O (1.4 Å) [69], respectively. The packing fraction (P.F.%) of the BST ceramics is illustrated in Table 2. As presented in Table 2, a lower *Q × f* value (8230 GHz) corresponds to a smaller P.F.% (71.69%), and a higher *Q × f* value (9519 GHz) correlates with a higher P.F.% (71.88%).

In addition, the oxygen vacancy defect in Ti-based microwave dielectric ceramics is widely acknowledged and is believed to be balanced through the generation of Ti^3+^ ions [49]. The primary reason for the higher dielectric losses is thought to be the Ti^3+^ ions in Ti-based ceramics. The mechanism could be represented by the following defect equation (Equations (5) and (6)) [50]:

(5) $$O_{O}^{\times }\to \frac{1}{2}O_{2}+V_{O}^{••}+2e^{\prime }\;$$

(6) $$Ti^{4+}+e^{\prime }\to Ti^{3+}\;$$

**Figure 7:**
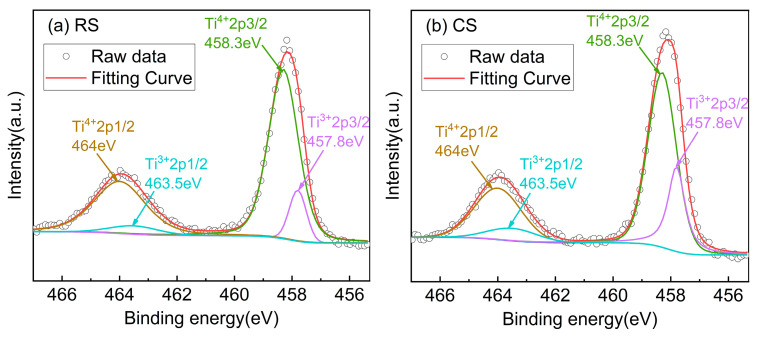
XPS spectra of BST ceramics: (**a**) Ti 2p for RS route sintered at 1400 °C (**b**) Ti 2p for CS route sintered at 1400 °C.

It is worth noting that the BST ceramics produced by the RS route at 1400 °C have a light yellow color, while those prepared by the CS route have a dark yellow color, as illustrated in Figure 6c. To explain this difference, valence states of Ti ions in BST ceramics prepared by the RS and CS routes were investigated by XPS analysis. In general, the two peaks of Ti 2p correspond to binding energies (B.E.) of approximately 458 (2p_3/2_) and 464 (2p_1/2_) eV [70]. Figure 7 presents the XPS spectroscopy of Ti 2p for BST samples prepared by the RS and CS routes at 1400 °C. The 2p_3/2_ and 2p_1/2_ region of Ti 2p in BST ceramics was fitted well to the Gaussian/Lorentzian sub-peak by means of the XPS-PEAK41 software, and the results are given in Figure 7 and Table 3. Table 3 presents that the relative peak area ratio of Ti^3+^ for the CS route (27.63%) is higher than the RS route (11.87%). This result suggests that the RS route is effective in suppressing the generation of trivalent titanium ions (Ti^3+^), which improves the quality factor (*tanδ*) of the BST ceramic. The combination of packing fraction (P.F.%) and XPS analysis explains the higher quality factor (lower dielectric loss) of the BST ceramics prepared by the RS route.

Table 2 shows that a smaller TCF value of −7.9 ppm/°C (1400 °C) for the RS route prepared ceramic sample is obtained compared to the TCF value of −12.1 ppm/°C (1400 °C) for the CS route. It was found that the TCF values of tungsten–bronze-structure Ba_6−3*x*_R_8+2*x*_Ti_18_O_54_ (R = Eu, Sm, Nd, Pr, and La) were generally influenced by additives, composition, and structural characteristics [71]. As all ceramic samples have no other extra additives and the unique composition Ba_4_Sm_28/3_Ti_18_O_54_, the key factor affecting their TCF may be the structural characteristics. Bond valence has been generally accepted as a useful tool for associating structural characteristics with TCF value. The bond valence (*S_jk_*) of the BST ceramics prepared by the RS and CS routes is calculated by means of Equations (7) and (8) [68]:

(7) $$S_{jk}=\sum_{k}s_{jk}\;$$

(8) $$s_{jk}=\exp\left[\frac{R_{jk}-d_{jk}}{0.37}\right]\;$$

where *d_jk_* represents the bond length, and *R_jk_* denotes the bond valence parameter. The TCF values and the calculated Ti-site bond valence (V_Ti_) of BST ceramics are presented in Table 2. Based on bond valence theory [47], the higher bond valence would lead to a higher restoring force of oxygen octahedrons, ultimately decreasing |TCF|. As shown in Table 2, the V_Ti_ of 4.136 v.u. for the RS route is higher than the 4.109 v.u. for the CS route, and the |TCF| of 7.9 ppm/°C for the RS route is smaller than the 12.1 ppm/°C for the CS route. As V_Ti_ increases, the |TCF| value decreases.

Microwave dielectric characteristics of high-k BST ceramics prepared by different routes are presented in Table 4 for comparison. Xu et al. [34] produced BST ceramics of *Q × f* = 11,300 GHz, and *ε_r_* = 80.8 by a sol-gel technique sintered at 1360 °C/3h. Guo et al. [35] prepared BST ceramics of TCF = −17.2 ppm/°C, *Q × f* = 10,099 GHz, and *ε_r_* = 81.2 via the SPS route sintered at 1200 °C/5min. Chen et al. [27] and Santha et al. [33] reported the microwave dielectric characteristics of BST ceramics fabricated by the CS route of TCF = −10.6 ppm/°C, *Q × f* = 9240 GHz, *ε_r_* = 81 and TCF = −12 ppm/°C, *Q × f* = 10,025 GHz, *ε_r_* = 76 were obtained, respectively. In the present work, excellent microwave dielectric characteristics (TCF = −7.9 ppm/°C, *Q × f* = 9519 GHz, *ε_r_* = 80.26) were achieved for Ba_4_Sm_28/3_Ti_18_O_54_ ceramics sintered at 1400 °C/4 h produced using the one-step RS route. Compared to other routes, the RS route without the calcination and secondary ball-milling processes is a straightforward, economical and effective method for preparing high-performance BST ceramics.

Far-infrared (FIR) reflection spectrum is commonly applied to evaluate intrinsic dielectric characteristics of electronic ceramics by means of the classic oscillator theory (Equation (9)) [72]:

(9) $$\varepsilon^{*}(\omega )=\varepsilon_{\infty }+\sum_{j=1}^{n}\varepsilon_{j}=\frac{\omega_{pj}^{2}}{\omega_{oj}^{2}-\omega^{2}-j\gamma_{j}\omega }$$

(10) $$R(\omega )={\left|\frac{1-\sqrt{\varepsilon^{*}(\omega )}}{1+\sqrt{\varepsilon^{*}(\omega )}}\right|}^{2}$$

where *γ_j_*, *ω_pj_*, *ε*_∞_, *ω_oj_*, and n are damping factor of the j-th mode, plasma frequency, optical frequency permittivity caused by electronic polarization, eigen frequency and sequence of phonon modes, individually. Equation (10) [72] illustrates the interrelation of complex permittivity (ε*(ω)) and reflectivity (*R(ω)*).

**Figure 8:**
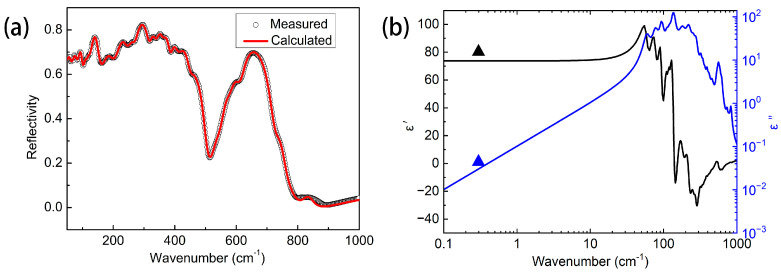
(**a**) Calculated and measured infrared reflection spectrum. (**b**) Fitted complex dielectric response of BST ceramic using RS route.

The room-temperature FIR spectrum of BST ceramics (50~1000 cm^−1^) sintered at 1400 °C using the RS route is illustrated in Figure 8a. The calculated FIR spectrum (red line) is consistent with the measured spectrum (black circle). Based on Kramers–Kronig (K-K) theory, the calculated FIR spectrum of BST ceramics prepared by the RS route is transformed into $\varepsilon^{\prime }(\omega )$ and $\varepsilon^{\prime \prime }(\omega )$, and this is presented in Table 5 and Figure 8b. Measured $\varepsilon^{\prime }(\omega )$ (black triangle) and $\varepsilon^{\prime \prime }(\omega )$ (blue triangle) are also shown Figure 8b. Compared to the theoretical permittivity value (43.27) calculated by the CM equation, the permittivity value (73.91) derived from the FIR spectrum is closer to the measured one (80.23), demonstrating that the dominant dielectric contribution of BST ceramics is phonon absorption. As can be seen in Table 5, the FIR spectrum of BST ceramics was fitted by the 24 individual modes. In particular, the eight infrared active modes (j = 1–8) below 220 cm^−1^ give the majority (84.07%) dielectric contribution to the permittivity. The *ε_∞_* value derived from Equation (10) is 4.69, only 6.3% of the total extrapolated permittivity (73.91), suggesting that ionic polarization plays an important role in the polarization that contributed to the *ε_r_* of BST ceramics.

## 4. Conclusions

For the first time, high-permittivity Ba_4_Sm_28/3_Ti_18_O_54_ ceramics with improved microwave dielectric characteristics have been successfully fabricated by the one-step RS route. The effects of various sintering routes (CS and RS routes) on the crystal structure, microstructure, valence states of Ti ions, and dielectric characteristics of BST ceramics have been investigated. Single-phase orthorhombic tungsten–bronze-structure and dense-microstructure BST ceramics were achieved using RS and CS routes at the optimum sintering temperature (1400 °C). The FIR spectrum analysis presents that the modes below 220 cm^−1^ give the majority (84.07%) dielectric contribution to the permittivity of BST ceramics prepared by the RS route. Compared to other routes, the RS route without the calcination and secondary ball-milling processes is a straightforward, economical and effective method for preparing high-performance BST ceramics.

## Figures and Tables

**Table 1:** The refinement parameters of Ba_4_Sm_28/3_Ti_18_O_54_ ceramics for RS and CS process.

Method	RS				CS			
S.T.	1300 °C	1350 °C	1400 °C	1450 °C	1300 °C	1350 °C	1400 °C	1450 °C
*a* (Å)	12.1524	12.548	12.1476	12.1513	12.156	12.1548	12.1596	12.1601
*b* (Å)	22.298	22.3001	22.2878	22.2946	22.307	22.3037	22.312	22.3134
*c* (Å)	7.6636	7.6637	7.659	7.6614	7.6612	7.6608	7.6633	7.6637
*V* (Å^3^)	2076.983	2077.279	2073.65	2075.567	2077.456	2076.841	2079.12	2079.458
*α = β = γ*	90	90	90	90	90	90	90	90
*R*_p_ (%)	5.86	6.26	6.03	6.37	5.79	5.94	6.00	6.14
*R*_wp_ (%)	7.58	8.04	7.62	8.23	7.44	7.69	8.01	8.37
*χ*^2^ (%)	1.38	1.6	1.45	1.71	1.55	1.59	1.81	2.04
*ρ_th_* (g/cm^3^)	5.883	5.882	5.893	5.886	5.88	5.884	5.875	5.876

**Table 2:** The measured microwave dielectric characteristics (*ε_r_*, *Q × f*, *tanδ* and TCF), and calculated theoretical permittivity (*ε_the_*), packing fraction (P.F.) and Ti-site bond valence of BST ceramics sintered at 1400 °C by RS and CS routes.

Method	Measured					Calculated		
	*ε_r_*	*Q* × *f* (GHz)	*f* (GHz)	*tanδ*	TCF (ppm/°C)	*ε_the_*	P.F. (%)	*V_Ti_* (v.u.)
RS (1400 °C)	80.26 ± 0.5	9519 ± 100	5.882	6.18 × 10^−4^ ± 0.07 × 10^−4^	−7.9 ± 1	43.27	71.88	4.136
CS (1400 °C)	79.38 ± 0.5	8230 ± 100	6.211	7.55 × 10^−4^ ± 0.09 × 10^−4^	−12.1 ± 1	41.65	71.69	4.109

**Table 3:** Binding energies and relative peak area ratio of Ti 2p spectra of BST ceramics using RS and CS routes.

Method	Binding Energy (eV)		Binding Energy (eV)		Relative Peak Area Ratio (%)
	Ti2p_1/2_		Ti2p_3/2_		Ti^3+^/(Ti^3+^ + Ti^4+^)
	IV	III	IV	III	
RS (1400 °C)	464	463.5	458.3	457.8	11.87%
CS (1400 °C)	464	463.5	458.3	457.8	27.63%

**Table 4:** Microwave dielectric characteristics of BST ceramics prepared by different method.

No	Method	S.T.	C.T.	*ε_r_*	*Q × f*	TCF	Ref.
		(°C)	(°C)		(GHz)	(ppm/°C)	
1	Sol–gel	1360 °C for 3 h	1000 °C for 3 h	80.8	10,099		[34]
2	Spark plasma sintering (SPS)	1200 °C for 5 min	1100 °C for 2 h	81.2	10,099	−17.2	[35]
3	Conventional solid-state (CS)	1360 °C for 3 h	1200 °C for 3 h	81	9240	−10.6	[27]
4	Conventional solid-state (CS)	1350 °C for 4 h	1175 °C for 4 h	76	10,025	−12	[33]
5	Conventional solid-state (CS)	1400 °C for 4 h	1150 °C for 3 h	79.38 ± 0.5	8230 ± 100	−12.1 ± 1	This work
6	Reaction sintering (RS)	1400 °C for 4 h	No calcining	80.26 ± 0.5	9519 ± 100	−7.9 ± 1	This work

**Table 5:** Phonon parameters obtained from the fitting of the infrared reflection spectrum of BST.

Mode	*ω_oj_*	*ω_pj_*	*γ_j_*	∆*ε_j_*
1	59.372	150.39	12.98	6.42
2	77.686	215.05	15.459	7.66
3	94.164	265.45	12.737	7.95
4	110.92	170.5	11.841	2.36
5	119.79	143.96	9.8071	1.44
6	136.27	549.38	20.392	16.3
7	180.78	608.44	47.303	11.3
8	220.41	651.18	40.424	8.73
9	254	397.26	33.883	2.45
10	276.38	393.1	29.563	2.02
11	321.14	233.55	25.539	0.529
12	342.62	193.1	25.398	0.318
13	364.24	135.29	24.327	0.138
14	389.39	158.91	22.899	0.167
15	415.22	210.92	44.179	0.258
16	455.19	129.34	31.593	0.0807
17	473.33	121.35	44.58	0.0657
18	533.19	212.21	39.59	0.158
19	559.52	378.82	41.725	0.458
20	584.43	267.57	42.129	0.21
21	617.56	238	43.317	0.149
22	732.06	95.576	42.165	0.017
23	776.86	82.733	65.188	0.0113
24	829.14	158.21	50.895	0.0364

*ε_∞_* = 4.69, *ε*_0_ = 69.22.

## Data Availability

The original contributions presented in the study are included in the article, further inquiries can be directed to the corresponding author.
